# Diagnostic Performance of First-Pass Myocardial Perfusion Imaging without Stress with Computed Tomography (CT) Compared with Coronary CT Angiography Alone, with Fractional Flow Reserve as the Reference Standard

**DOI:** 10.1371/journal.pone.0149170

**Published:** 2016-02-19

**Authors:** Kazuhiro Osawa, Toru Miyoshi, Takashi Miki, Yasushi Koyama, Shuhei Sato, Susumu Kanazawa, Hiroshi Ito

**Affiliations:** 1 Department of Cardiovascular Medicine, Okayama University Graduate School of Medicine, Dentistry and Pharmaceutical Sciences, Okayama, Japan; 2 Department of Cardiology, Sakurabashi Watanabe Hospital, Osaka, Japan; 3 Department of Radiology, Okayama University Graduate School of Medicine, Dentistry and Pharmaceutical Sciences, Okayama, Japan; Scuola Superiore Sant'Anna, ITALY

## Abstract

Coronary computed tomography angiography (CCTA) in combination with first-pass CT myocardial perfusion imaging (MPI) has a better diagnostic performance than CCTA alone, compared with invasive coronary angiography as the reference standard. The aim of this study was to investigate the additional diagnostic value of first-pass CT-MPI without stress for detecting hemodynamic significance of coronary stenosis, compared with invasive fractional flow reserve (FFR). We recruited 53 patients with suspected coronary artery disease undergoing both CCTA and first-pass CT-MPI without stress and invasive FFR, and 75 vessels were analyzed. We used the same raw data for CCTA and CT-MPI. First-pass CT-MPI was reconstructed by examining the diastolic signal densities as a bull’s eye map. Invasive FFR <0.8 was considered as positive. On per-vessel analysis, the area under the receiver operating characteristic curve for CCTA plus first-pass CT-MPI and CCTA alone was 0.81 (0.73–0.90) and 0.70 (0.61–0.81), respectively (*P* = 0.036). CCTA plus first-pass CT-MPI without stress showed 0.73 sensitivity, 0.74 specificity, 0.53 positive predictive value, and 0.87 negative predictive value for detecting hemodynamically significant coronary stenosis. First-pass CT-MPI without stress correctly reclassified 38% of CCTA false-positive vessels as true negative. First-pass CT-MPI without stress combined with CCTA demonstrated excellent diagnostic accuracy, compared with invasive FFR as the reference standard. This technique could complement CCTA for diagnosis of coronary artery disease.

## Introduction

Coronary computed tomography angiography (CCTA) is an excellent method for detecting coronary artery disease (CAD), although it is still challenging in cases with heavy calcification and intermediate stenotic lesions.[1] First-pass CT myocardial perfusion imaging (MPI) without stress is simultaneously obtained from the same raw data used for CCTA and requires no additional contrast medium and radiation exposure. We have recently reported the usefulness of first-pass CT-MPI without stress in combination with CCTA for diagnosis of obstructive CAD, compared with CCTA alone, with invasive coronary angiography (ICA) as the reference standard.[2] Invasive coronary angiography is a gold standard for anatomical detection of obstructive CAD, although it has limited capacity to determine the hemodynamic significance of stenosis, which is determined by decreased fractional flow reserve (FFR).[3, 4]

Measurement of FFR by ICA is the gold standard for diagnosis of coronary stenosis causing lesion-specific ischemia.[5] The FAME (Fractional Flow Reserve vs Angiography for Multivessel Evaluation) study has shown that invasive FFR-guided decisions about revascularization improve event-free survival compared with coronary angiography-guided decisions alone.[6] Consequently, FFR is the accepted reference standard for assessing the functional significance of CAD in a lesion-specific manner.[7] The use of FFR, however, is inherently limited by its invasiveness and costs. Moreover, FFR cannot always be measured in vessels owing to extreme tortuosity and/or coronary calcification. These issues underscore the need for more accurate noninvasive diagnostic tests for gatekeeping to the catheterization laboratory.

The purpose of this study was to investigate the diagnostic accuracy of first-pass CT-MPI without stress in combination with CCTA to detect coronary lesions causing myocardial ischemia, compared with invasively determined FFR as the reference standard.

## Materials and Methods

### Study design and patient population

We prospectively enrolled patients with suspected or known CAD referred for coronary angiography and FFR measurement at Okayama University Hospital between November 2012 and February 2015. CCTA was performed within 60 days before invasive coronary studies. Invasive FFR was performed on coronary arteries with luminal diameter reduction between 30% and 90% in a vessel segment ≥2 mm in diameter according to CCTA. We excluded patients with previous coronary artery intervention or coronary bypass surgery, contraindications to iodinated contrast medium, adenosine, β-blocking agents and nitroglycerin, and presence of Q waves on resting electrocardiography (ECG).

### Ethics statement

The study was approved by the Ethics Committee of Okayama University Graduate School of Medicine, Dentistry, and Pharmaceutical Sciences (Okayama, Japan). This study was conducted according to the principles expressed in the Declaration of Helsinki. All patients provided written informed consent to be included in the study.

### Image acquisition

CT scans were performed using a 128-slice CT scanner (SOMATOM Definition Flash; Siemens Medical Solutions, Erlangen, Germany) with the following parameters: detector collimation, 64 × 0.6 mm, equaling a slice acquisition of 128 × 0.6 mm using the flying focal spot technique; table pitch was adapted to heart rate (0.17–0.38); rotation time, 275 ms; tube current–time product, 360 mAs; and tube voltage, 120 kV with use of retrospective electrocardiogram-triggered spiral acquisition. All of the patients arrived at the hospital 1 h before the scheduled CT scanning time, and those with a persistent high heart rate ≥60 beats/min received oral metoprolol (20–40 mg). If the heart rate did not sufficiently decrease to <60 beats/min before CT, the patients received intravenous landiolol hydrochloride (0.125 mg/kg) until the heart rate was <60 beats/min. A test CT acquisition was performed at the level of the ascending aorta after administration of 10 mL Omnipaque 350 contrast medium (Daiichi Sankyo, Japan) followed by 20 mL saline. Low-dose images were obtained every 1 s. The delay before the formal scan was calculated as the time to peak enhancement in the ascending aorta plus 5 s to ensure enhancement of the ventricular region. For the final scan, the contrast agent was injected for 12 s, followed by a second bolus of contrast medium that was diluted to 50% and injected for 8 s, and then a chaser bolus of saline for 5 s. In all cases, the flow rate was calculated as body weight × 0.07 mL/s.[2]

### Image processing and interpretation

#### Reference standard: FFR

FFR ≤0.80 indicated significant coronary lesions. FFR measurement was performed according to standard practice.[8]

#### Coronary CTA

The presence of significant stenotic lesions on CCTA was defined as luminal narrowing >50% and nonevaluable with CCTA owing to heavy calcification.

#### CT-MPI without stress

First-pass CT-MPI without stress was analyzed with a commercially available cardiac evaluation software program (Cardiac CT Image Report CUR-SV03, bundled software Vascular Volume Mapping, Siemens compatible version 1.0; Argus B.M.C., Ehime, Japan). Retrospective image reconstruction was performed at 5% phase increments throughout the cardiac cycle. A total of 20 image data sets were reconstructed. The slice was 3-mm thick. The short-axis images were reconstructed from 20 image data sets using the standard double oblique method and were finally expressed in a bull’s eye map. Assignment of the left ventricular segment was based on the 16 myocardial segment model, excluding the apical segment [9]. We evaluated variation in myocardial enhancement during the diastolic phase. CT-MP images were expressed by color maps on the basis of the CT values of the left ventricular myocardium. Warm colors represented hyperenhanced areas with high CT values and cold colors represented hypoenhanced areas with low CT values. From the tone of the cold colors and ratio of the cold color area, the hypoenhanced areas were graded on a three-point scale of mild, moderate and severe. When first-pass CT-MPI without stress showed moderate or severe perfusion attenuation, corresponding vessels were considered to have significant stenosis. Image reading was performed by a cardiologist and radiologist who were blinded to CCTA findings. Disagreements in data analysis between the two observers were resolved by consensus reading.

#### Radiation dose estimates of CT

We calculated the effective radiation dose for each component of the cardiac CT examination by the dose–length product.

### Statistical analysis

**Fig 1:**
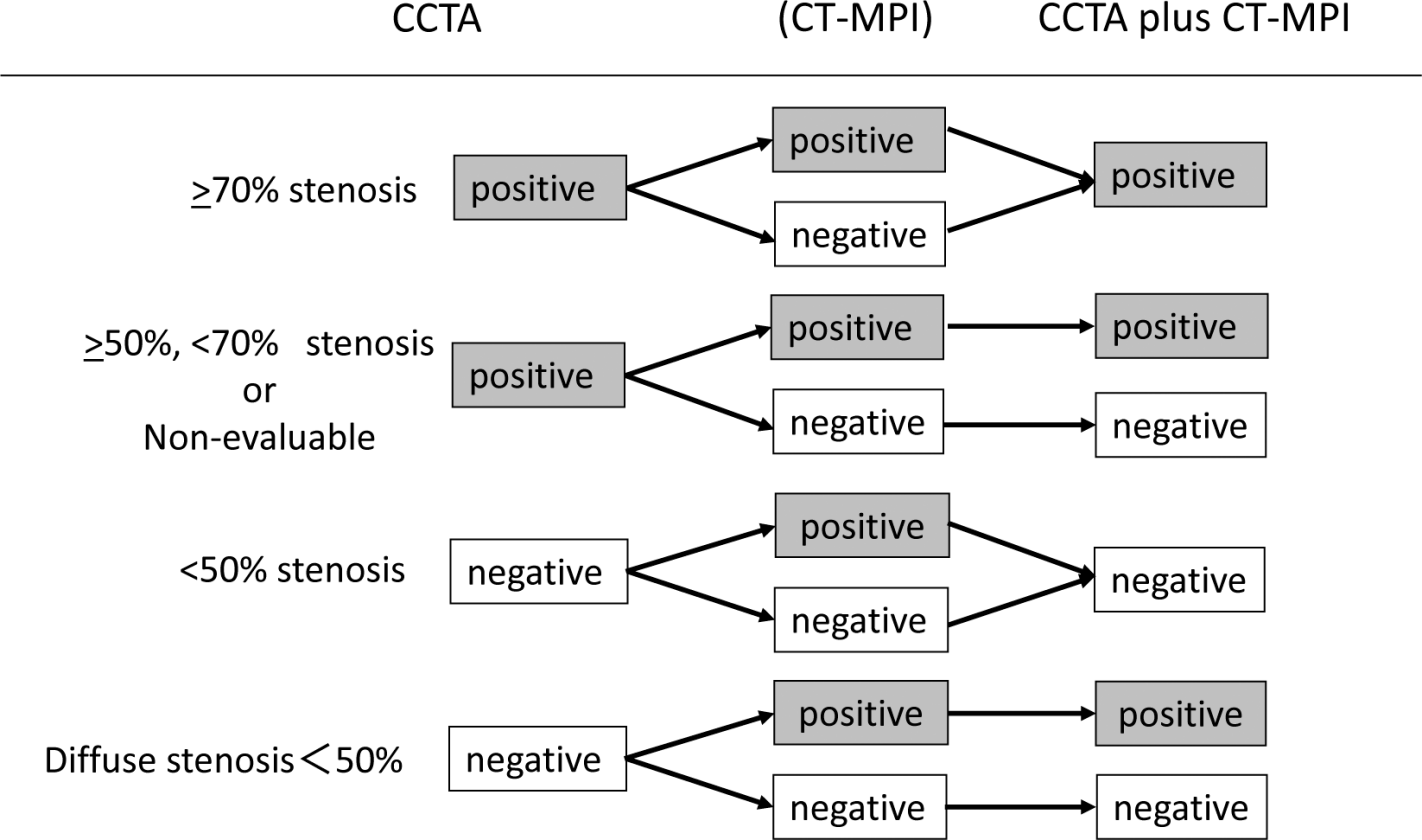
Reclassification criteria. Before CT-MPI analysis, nonevaluable with CCTA was defined as positive for stenosis using the following criteria: those with no vessel wall definition owing to marked motion artifacts or heavy calcification that precluded acquisition of diagnostic information.

With FFR as the reference standard, the diagnostic accuracy of CCTA and CCTA plus CT-MPI without stress was expressed in terms of accuracy, sensitivity, specificity, positive predictive value and negative predictive value for the detection of vascular territories with significant obstructive coronary artery stenosis. Diagnostic performance was calculated on a vessel basis. CCTA diagnosis was reclassified according to CT-MPI without stress. After CT-MPI analysis, nonevaluable vessels with CCTA were considered positive for stenosis only if they corresponded to a CT-MPI defect in the same vascular distribution. Moderate stenosis (50–70%) on CCTA were reclassified as negative if CT-MPI showed no defect in the same distribution. Diffuse stenosis with luminal narrowing of 30–50% on CCTA was reclassified as positive if CT-MPI showed a defect in the same distribution. CCTA stenosis was not reclassified when no stenosis <50% or >70% was apparent on CCTA (Fig 1). The area under the receiver operating characteristic curve (C statistic) was calculated for all diagnostic testing strategies for which a reference standard was available. A value of *P* < 0.05 was considered statistically significant. The area under the receiver operating characteristic curve was compared using the ROCCOMP command (Stata version 10; Stata Corp., College Station, TX, USA).

## Results

We analyzed 75 vessels from 53 participants (36 men, age 71.4±7.8 years) (Table 1). The mean body mass index was 23.0 ± 3.5 kg/m^2^ and the Agatston score was 481 (996), expressed as the median (interquartile rage). The amount of CT contrast medium was 73 ± 12 mL and the dose–length product for cardiac CT examination was 1399.4 ± 314.2 mGy-cm (19.6 ± 4.4 mSV). The average infusion dose of adenosine for FFR measurement was 157 ± 12 μg/kg/min.

**Table 1:** Patients’ characteristics.

	total (n = 53)
Age (years)	71.4 ± 7.8
Male sex, n (%)	36 (68)
Diabetes mellitus, n (%)	36 (68)
Hypertension, n (%)	43 (81)
Dyslipidemia, n (%)	36 (68)
Body mass index (kg/m^2^)	23.0±3.5
Current smoking, n (%)	9 (17)
Agatston score	481 (996)
Medications	
ACEI/ARB, n (%)	32 (60)
Calcium channel blocker, n (%)	27 (51)
Beta blocker, n (%)	12 (23)
Statin, n (%)	20 (38)
Agatston score	481 [236–1282]
Target vessel (n = 75): LAD / LCX / RCA, n (%)	43 (57) / 17(23) / 15(20)
Number of significant stenosis in target vessel*	1.27 ± 0.45

Data are the number (%) or mean ± SD or median [25th–75th percentile]. ACEI, angiotensin-converting enzyme inhibitor; ARB, angiotensin II receptor blocker.

*Significant stenosis indicates vessel with luminal narrowing >50% and/or significant calcification determined with CCTA.

**Fig 2:**
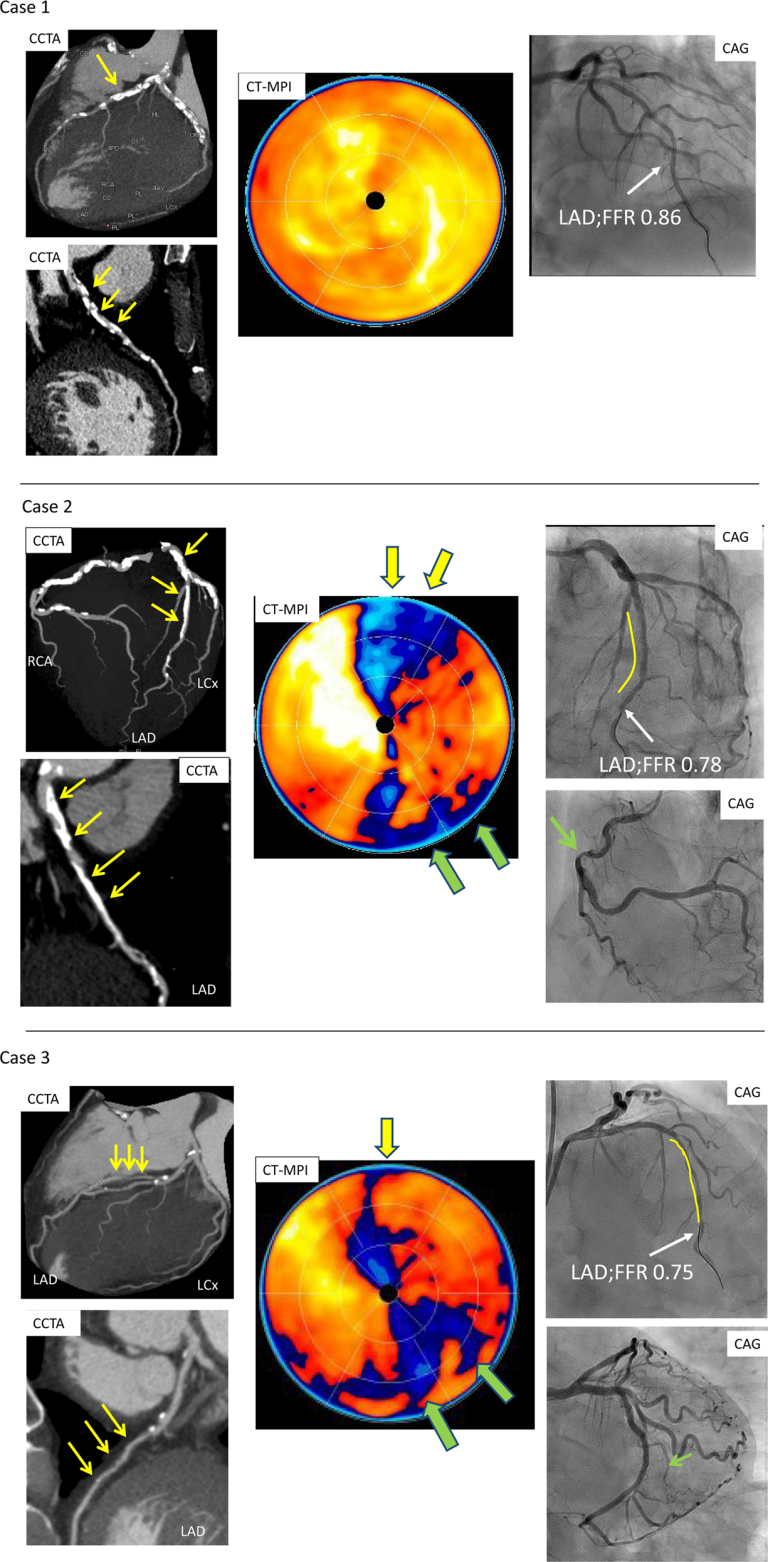
Case examples of CCTA, CT-MPI without stress, coronary angiography, and invasive FFR. Case 1. CCTA showed significant coronary artery calcification in the proximal LAD (yellow arrow). CT-MPI without stress showed no perfusion defect. Coronary angiography confirmed no significant stenosis in the LAD with invasive FFR = 0.86. Case 2. CCTA showed significant coronary artery calcification in the left main trunk and proximal LAD (yellow arrow). CT-MPI without stress showed perfusion defects in the anterior (yellow arrow) and inferior (green arrow) walls. Coronary angiography confirmed 58% luminal stenosis in the LAD with invasive FFR = 0.78 (yellow line) and 56% stenosis in the RCA (green arrow). Case 3. CCTA showed diffuse stenosis <50% in the middle of the LAD (yellow arrow). CT-MPI without stress showed perfusion defects in the anterior (yellow arrow) and inferolateral (green arrow) walls. Coronary angiography confirmed diffuse 55% stenosis in the LAD with invasive FFR = 0.75 (yellow line) and 69% of luminal stenosis in the LCx. LCx, left circumflex artery; RCA, right coronary artery.

Representative images are shown in Fig 2. With invasive FFR as the reference standard of this study, 22 of the 75 (29%) vessels being stenotic was identified as significant stenosis. Among the 22 stenotic lesions, 18 (82%), one (5%), and three (13%) were in the left anterior descending artery (LAD), left circumflex artery, and right coronary artery, respectively. Comparison of CCTA alone and CCTA plus first-pass CT-MPI without stress was performed (Table 1). CCTA identified 43 (57%) significant stenotic vessels, including 18 (42%) with luminal narrowing >70%, 14 (33%) with moderate stenosis (50–70%), and 11 (26%) with severe calcification. There were six vessels with diffuse stenosis (30–50%), which were considered as nonsignificantly stenotic vessels on CCTA. First-pass CT-MPI without stress identified 30 (40%) vascular territories with perfusion abnormalities. CCTA plus first-pass CT-MPI without stress identified 35 (47%) significantly stenotic vessels.

**Fig 3:**
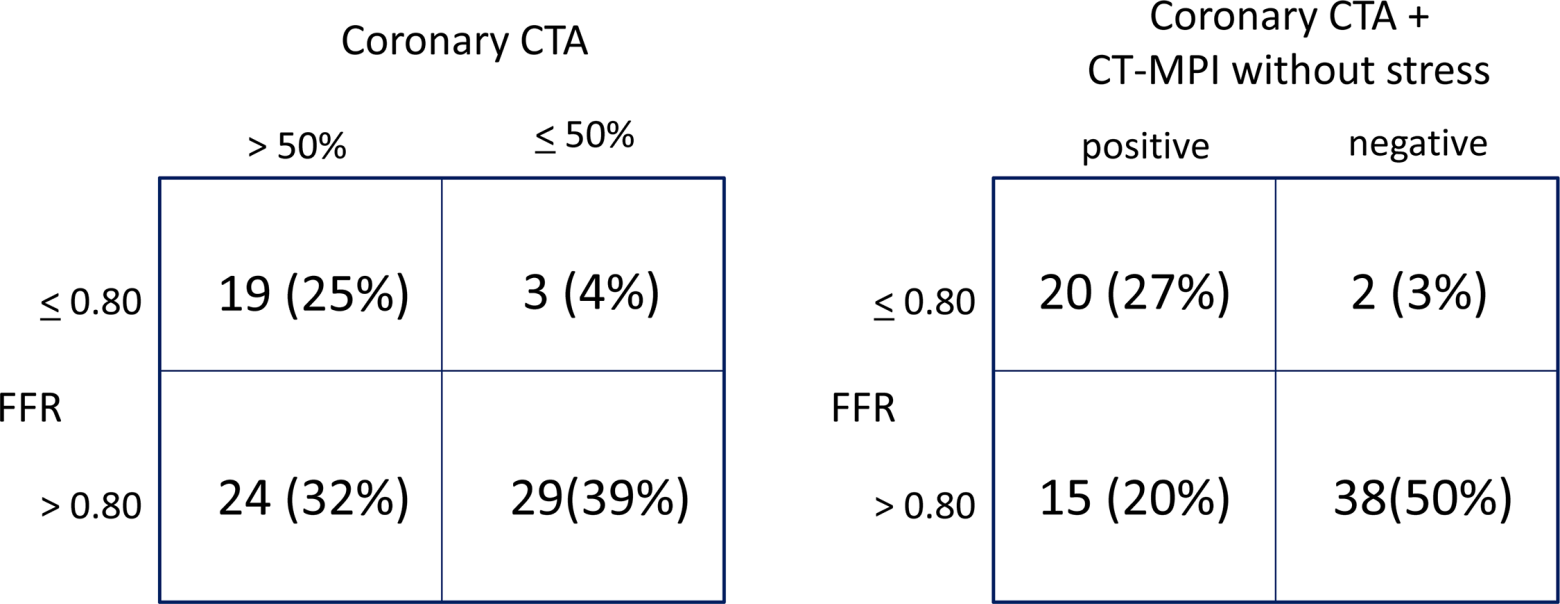
Agreement for detection of ischemia (FFR ≤0.80) between CCTA and CCTA + CT-MPI without stress on a per-vessel basis. Data given as number (%); *n* = 75 vessels.

According to the reclassification criteria shown in Fig 1, with CCTA plus first-pass CT-MPI without stress, four vessels with severe calcification and eight with intermediate stenosis (50–70%) were reclassified as nonsignificantly stenotic vessels from significantly stenotic vessels, and four vessels with diffuse stenosis (30–50%) were reclassified as significantly stenotic vessels form nonsignificantly stenotic vessels. First-pass CT-MPI without stress reclassified 38% patients from false negative into true negative. Consequently, first pass CT-MPI without stress correctly reclassified 38% of CCTA false-positive vessels as true negative (Fig 3). Diagnostic accuracy of CCTA plus first-pass CT-MPI on per-vessel analysis was significantly greater than that of CCTA alone (Table 2). We observed a significant improvement of C statistics of CCTA plus first-pass CT-MPI without stress compared with CCTA alone, with an increase from 0.71 to 0.82 (*P* = 0.036).

**Table 2:** Diagnostic accuracy of CCTA and CCTA plus CT-MPI without stress for detection of significantly stenotic coronary arteries in 75 vessels.

	CCTA	CCTA plus CT-MPI without stress
Number of vessels		
True positive	19	20
False negative	3	2
False positive	24	15
True negative	29	38
Accuracy (%)	64	77
Sensitivity (%)	86	91
Specificity (%)	55	72
Positive predictive value (%)	44	57
Negative predictive value (%)	91	95
C statics	0.71 (0.61–0.81)	0.82 (0.73–0.90)

We also performed analysis with different reclassification criteria, because the decision to carry our CCTA plus first-pass CT-MPI without stress in vessels with luminal stenosis >70% was based on the results of CCTA, not first-pass CT-MPI. In this analysis, diagnostic accuracy, sensitivity, specificity, positive predictive value and negative predictive value for CCTA plus first-pass CT-MPI without stress on a per-vessel basis were 81%, 82%, 81%, 64%, and 91%, respectively. C statistics of CCTA plus first-pass CT-MPI without stress compared with CCTA alone tended to increase from 0.71 (95% confidential interval: 0.61–0.81) to 0.81 (0.72–0.91) (*P* = 0.08)

## Discussion

This study demonstrated the usefulness of first-pass CT-MPI without stress in addition to CCTA for detecting coronary stenosis causing myocardial ischemia as the reference standard of invasive FFR in patients without a history of CAD. First-pass CT-MPI without stress has the potential for incremental diagnostic value for noninvasive assessment of hemodynamically significant CAD.

We have recently reported the additional diagnostic value of first-pass CT-MPI without stress for detecting significant luminal narrowing of the coronary artery assessed with ICA, which was the reference standard. A decrease in myocardial signal density identified with first-pass CT-MPI without stress means that myocardial vascular volume is impaired because of significant stenosis in the corresponding coronary artery, which reflects detection of myocardial ischemia under conditions of hyperemia. However, morphological luminal narrowing of the coronary artery does not always cause myocardial ischemia [10]. In the present study, we adopted invasive FFR as the reference standard. FFR is known as a gold standard for functional assessment of coronary artery stenosis for coronary revascularization. The clinical utility of FFR for deciding whether coronary artery stenosis requires revascularization has been described previously [6, 11]. ICA still plays an important role in diagnostic algorithms for CAD. Coronary angiography is now performed with safety, but serious complications such as coronary dissection, stroke, and myocardial infarction cannot be avoided completely [12]. The reduction in false-positive rate by first-pass CT-MPI without stress could decrease unnecessary invasive cardiac catheterization and could be a gatekeeper to ICA and revascularization.

First-pass CT-MPI without stress is especially useful in analysis of coronary arteries with significant calcification or intermediate stenosis. The use of dual-energy CT or subtraction method is a recent technical advance in CT technology, which could overcome the problem of coronary artery calcification [13, 14]. It is difficult to evaluate whether intermediate coronary artery stenosis has really caused myocardial ischemia [15]. Transluminal attenuation gradient is also one of the additional techniques for detecting coronary lesions causing myocardial ischemia [16]. Furthermore, emerging evidence demonstrates that FFR-CT could improve diagnostic accuracy of intermediate stenosis [17, 18]. Recent studies have demonstrated that pharmacological adenosine stress CT-MPI is a promising method for detection of CAD [19]; however, it needs additional contrast medium and radiation exposure. First-pass CT-MPI without stress is expressed as a bull’s eye map, which could be an easy-to-understand format for general physicians. In addition, first-pass CT-MPI does not require additional radiation doses or contrast medium use. Thus, first-pass CT-MPI has advantages, while the best gold standard would have been the documentation of coronary stenosis at ICA and coherent inducible myocardial ischemia at a different perfusion or wall motion imaging stress test. First-pass CT-MPI should be used as an adjunct to the diagnosis of ischemia-causing coronary stenosis.

Recently, myocardial perfusion CT has emerged for the detection of myocardial ischemia [19–21]. Myocardial perfusion CT shows reasonable accuracy for the detection of myocardial ischemia compared with invasive FFR measurement [22]. Perfusion CT allows visualization of myocardial contrast enhancement, thus, both visual and quantitative analysis of myocardial perfusion is possible [23]. However, validation of other potential quantitative parameters, such as myocardial attenuation in rest and stress phases, or the difference in attenuation between the stress and rest phases, has not been fully evaluated. In contrast, FFR derived from CCTA (FFR-CT) enables noninvasive assessment of the hemodynamic significance of coronary artery lesions and coupling of the anatomic severity of coronary stenosis with its physiological effects [24]. At present, several multicenter clinical trials of FFR-CT have been completed and have shown a generally high diagnostic performance of FFR-CT [17, 25–28]. However, whether FFR-CT can more effectively improve clinical outcomes remains unknown.

The present study had several limitations. First, FFR evaluation was not possible in all coronary arteries and 82% of the evaluated lesions were in the LAD in this study, which could have led to verification bias in the results. Second, the number of subjects was small, given that this study was performed in a single institution. Patients with suspected CAD were recruited, and ICA was presumably prompted by CCTA in some cases. Therefore, patients were highly selected and further studies with a larger group are needed. Third, the amount of radiation exposure in this study was a concern. Our protocol adopted retrospective ECG gating image acquisition and could be improved by ECG-control modulation scanning. Another effective method for dose reduction is using lower tube voltages. Even though reducing the tube voltage increases image noise and can result in degradation of image quality, appropriate reconstruction algorithms may be helpful for further substantial dose reduction. Fourth, β receptor blockers and sublingual nitroglycerin were used before CT data acquisition in all participants. Acquiring perfusion at rest under β receptor blockers may underestimate perfusion impairment due to reduction of cardiac work. Sublingual nitroglycerin may make attenuated perfusion smaller [29]. These medications are generally essential for acquiring evaluable images. Thus, the implications of the use of cardiac medications on myocardial perfusion need careful consideration. Fifth, patients with acute coronary syndromes and previous coronary intervention or bypass surgery were excluded from the present study. Thus, generalizability of first-pass CT-MPI without stress to these specific populations of patients with CAD is unknown.

In conclusion, first-pass CT-MPI without stress provides incremental value over CCTA for evaluating coronary lesions causing myocardial ischemia. The combination of CCTA plus first-pass CT-MPI without stress may be useful for comprehensive non-invasive anatomical and functional assessment of CAD.
